# Efficacy of immune checkpoint inhibitors combined with different chemotherapy in recurrent pancreatic cancer: a retrospective study

**DOI:** 10.3389/fimmu.2026.1841783

**Published:** 2026-07-30

**Authors:** Qiang Tao, Lingmin Jiang, Ruiqi Wang, Xin Huang, Pu Xi, Jun Wang, Shengping Li, Chaobin He

**Affiliations:** 1 State Key Laboratory of Oncology in South China, Guangdong Provincial Clinical Research Center for Cancer, Collaborative Innovation Center for Cancer Medicine, Sun Yat-sen University Cancer Center, Guangzhou, PR, China; 2 Department of Hepatobiliary and Pancreatic Surgery, The Eighth Affiliated Hospital, Sun Yat-sen University, Shenzhen, China; 3 Department of Thyroid and Hernia Surgery, Guangdong Provincial People’s Hospital (Guangdong Academy of Medical Sciences), Southern Medical University, Guangzhou, Guangdong, China; 4 Department of Pancreatobiliary Surgery, Sun Yat-sen University Cancer Center, Guangzhou, PR, China; 5 Department of Head and Neck Surgery, The Affiliated Cancer Hospital of Zhengzhou University & Henan Cancer Hospital, Zhengzhou, China

**Keywords:** AG, FOLFIRINOX, immune checkpoint inhibitors, pancreatic cancer, recurrence, survival

## Abstract

**Objective:**

Recurrence affects 65%-75% of pancreatic cancer (PC) patient post-resection. Established salvage strategies for recurrent PC are lacking, and systemic chemotherapy offers limited survival benefits. This study aimed to evaluate the efficacy and safety of immune checkpoint inhibitors (ICIs) combined with different chemotherapy in patients with recurrent PC, specifically FOLFIRINOX (a combination of 5-fluorouracil, leucovorin, irinotecan, and oxaliplatin) and Gemcitabine plus Nab-paclitaxel (AG).

Materials and methods: We retrospectively screened 201 patients with recurrent PC between March 2016 and September 2022. A total of 113 eligible patients were enrolled, including 23 in the chemotherapy combined with ICIs group and 90 in the chemotherapy-only group. The primary endpoint was overall survival (OS), and secondary endpoints included progression-free survival (PFS). Subgroup analyses were performed based on chemotherapy regimens (FOLFIRINOX vs. AG).

**Results:**

In the overall cohort, the chemotherapy combined with ICIs group demonstrated a trend toward improved survival compared to the chemotherapy group. Subgroup analysis revealed that among patients receiving the FOLFIRINOX regimen, the addition of ICIs was significantly associated with improved OS (median OS: not reached vs. 14.9 months; HR = 0.266 [95% CI: 0.077-0.919]; log-rank *P* = 0.025; Cox *P =* 0.036) and showed a superior trend in PFS (*P* = 0.080) compared to FOLFIRINOX alone. Conversely, no significant survival benefit was observed in the AG subgroup (*P* > 0.05).

**Conclusion:**

Combining ICIs with FOLFIRINOX-based chemotherapy was significantly associated with improved OS in patients with recurrent PC with an acceptable safety profile. This combination strategy represents a promising therapeutic option for recurrent PC, particularly when paired with the FOLFIRINOX backbone.

## 1 Introduction

Pancreatic cancer (PC) is a highly aggressive malignancy of the digestive system and remains one of the most lethal tumors worldwide (1). Characterized by its insidious onset and rapid progression, the 5-year overall survival rate for PC remains as low as 13% (2). Although curative-intent surgery offers a potential chance of long-term survival for a small proportion of patients diagnosed at an early stage, the majority still face a substantial risk of local recurrence or distant metastasis after resection (3, 4). At present, there is no standardized consensus for the management of recurrent PC, and salvage treatment largely relies on systemic chemotherapy (5).

Gemcitabine plus nab-paclitaxel (AG) and FOLFIRINOX are currently the two major first-line chemotherapy regimens for advanced pancreatic cancer (6). However, when these conventional high-intensity regimens are directly applied to recurrent pancreatic cancer, the objective response rate (ORR) is generally unsatisfactory and the survival benefit remains limited (7). Moreover, because of impaired physical condition and cumulative toxicities resulting from prior surgery and adjuvant treatment, patients with recurrent disease often exhibit reduced tolerance to systemic chemotherapy. Therefore, the development of more effective and less toxic combination strategies has become a major unmet clinical need in the management of recurrent PC.

In recent years, immune checkpoint inhibitors (ICIs) have shown remarkable antitumor activity in multiple solid tumors (8, 9), yet their efficacy as monotherapy in PC has been disappointing (10). This is largely attributed to the uniquely immunosuppressive tumor microenvironment (TME) and the dense desmoplastic stroma, both of which severely restrict the infiltration and function of effector T cells (11, 12). Notably, emerging evidence on the molecular evolution of PC has revealed substantial biological differences between recurrent lesions and treatment-naive primary tumors (13, 14). Under the selective pressure imposed by prior surgery and systemic therapy, recurrent tumors often undergo a pronounced genetic bottleneck, characterized by an increased mutational burden, enhanced subclonal heterogeneity, and aberrant activation of key signaling pathways such as MAPK/ERK and PI3K/AKT (15). This treatment-driven genetic remodeling and clonal evolution suggest that recurrent PC may harbor more neoantigens and thus possess greater intrinsic immunogenic potential than newly diagnosed disease, providing a biological rationale for immunotherapeutic intervention in the recurrent setting (16, 17).

In addition, chemoimmunotherapy has attracted increasing attention in recent years. Beyond its direct cytotoxic effects, chemotherapy can remodel the local immune microenvironment by inducing immunogenic cell death (ICD) and promoting neoantigen release, thereby potentially enhancing the efficacy of immunotherapy (18). Importantly, previous studies have often treated systemic chemotherapy as a single, uniform intervention, while overlooking the heterogeneity among different chemotherapeutic regimens in their ability to synergize with immunotherapy (19). For example, oxaliplatin, a key component of the FOLFIRINOX regimen, has been shown to possess strong immunomodulatory properties and a robust capacity to induce ICD (20), whereas the AG regimen may differ substantially in its mechanisms of immune activation (21). However, whether these two chemotherapy regimens, when combined with ICIs, can improve outcomes in recurrent PC remains unclear.

Therefore, this retrospective study focused on patients with recurrent PC and systematically evaluated and compared the efficacy and safety of chemotherapy plus immunotherapy and chemotherapy with the aim of providing clinically relevant and potentially translational insights for the precision management of recurrent PC.

## 2 Patients and methods

### 2.1 Study design and patients

This retrospective study protocol was approved by the Institutional Review Board of Sun Yat-sen University Cancer Center. We screened a total of 201 patients who experienced their first recurrence of PC after R0 resection between March 2016 and September 2022. To be eligible for inclusion, patients had to meet the following criteria: (1) pathologically verified pancreatic ductal adenocarcinoma post-radical surgery; (2) recurrent PC confirmed via multidisciplinary assessment using advanced imaging modalities (contrast-enhanced CT, MRI, or PET-CT); (3) age ≥ 18 years with an Eastern Cooperative Oncology Group performance status (ECOG PS) of ≤ 2; (4) Child-Pugh grade A or B; and (5) the presence of at least one target lesion measurable.

**Figure 1:**
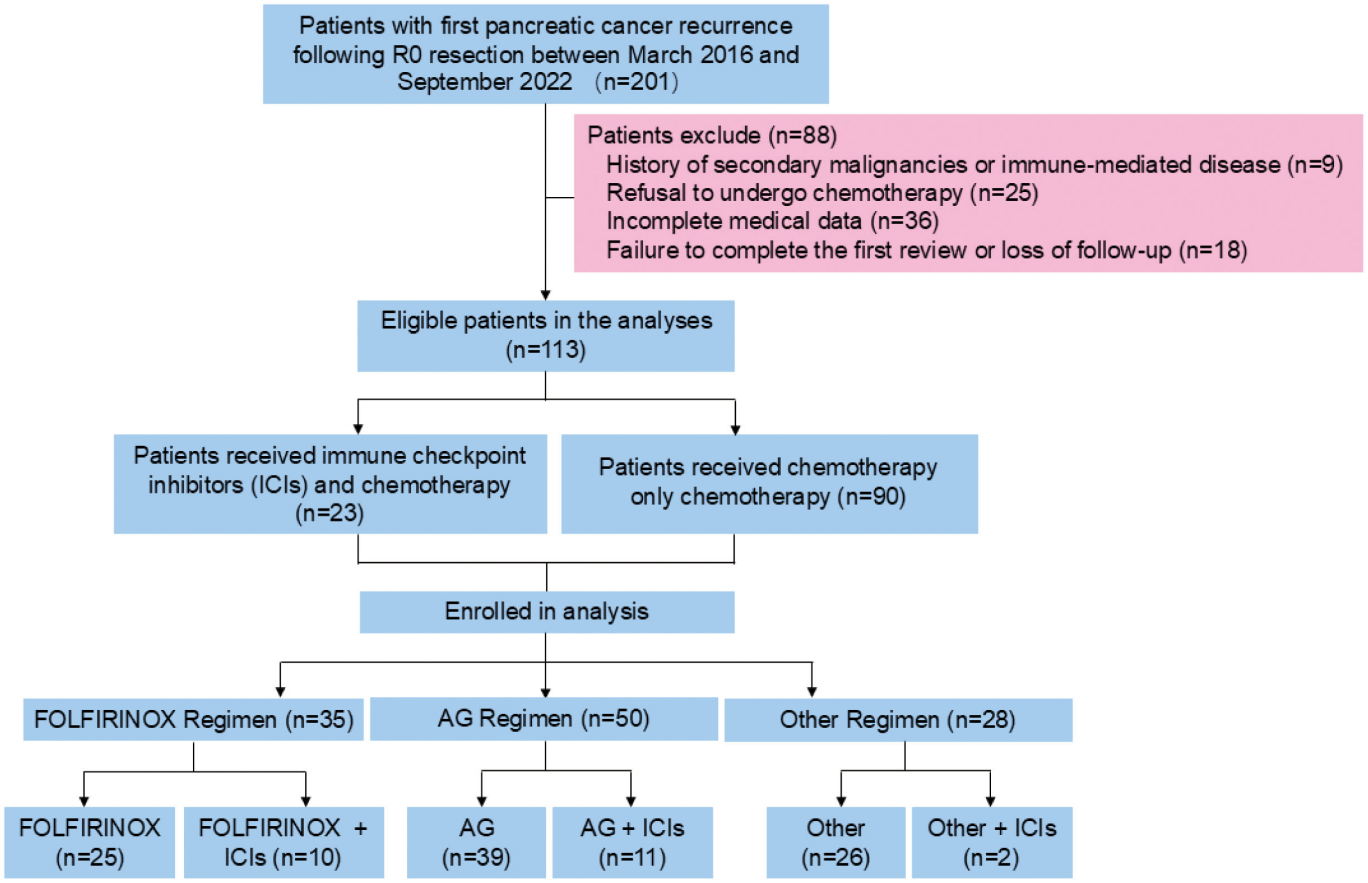
Flow diagram for patient selection.

As depicted in the study flowchart (**Figure 1**), among the 201 screened individuals, 88 were excluded based on the following predefined criteria: (1) co-occurrence of other primary malignancies or pre-existing immune-mediated disorders (n = 9); (2) elective refusal of cytotoxic chemotherapy (n = 25); (3) insufficient clinical or pathological data (n = 36); and (4) absence of the baseline treatment response assessment or loss to follow-up (n = 18). Consequently, a final cohort of 113 eligible patients was enrolled for subsequent analysis.

### 2.2 Treatment regimen

All enrolled patients received systemic chemotherapy as their first-line treatment initiated at the time of documented disease recurrence, categorized into three primary regimens based on clinical guidelines and patient tolerance. The FOLFIRINOX (5-fluorouracil, leucovorin, irinotecan, and oxaliplatin) regimen constituted a sequential administration of oxaliplatin via a 2-hour intravenous infusion, followed by leucovorin (200 mg/m^2^) over 2 hours. Subsequently, irinotecan (180 mg/m^2^) was delivered as a 90-minute intravenous infusion starting 30 minutes after leucovorin. The cycle concluded with a 5-fluorouracil (5-FU) component, comprising an initial intravenous bolus (400 mg/m^2^) and a subsequent 46-hour continuous infusion (2400 mg/m^2^) (22). The AG (Gemcitabine plus Nab-paclitaxel); regimen consisted of nab-paclitaxel (125 mg/m^2^) administered as a 30-minute intravenous infusion, followed by intravenous gemcitabine (1000 mg/m^2^) on days 1, 8, and 15 of each cycle (23). Other treatment regimens primarily consisted of S-1-based chemotherapy, including S-1 monotherapy and the SOXIRI regimen (a combination of S-1, oxaliplatin, and irinotecan). Other chemotherapy group was defined as regimens consisting primarily of S-1-based chemotherapy, which specifically included S-1 monotherapy and the SOXIRI regimen. S-1 monotherapy was administered orally in 21-day cycles, consisting of 14 consecutive days of treatment followed by a 7-day rest period. The SOXIRI regimen consisted of oxaliplatin (85 mg/m2) for 2h intravenously and irinotecan (150 mg/m2) for 90 min intravenously. S-1 (80 mg/m2) was orally administered twice daily, every other day for 2 weeks (24–26). The dose of S-1 was determined to body surface area and institutional practice. Patients received S-1 twice daily. ICIs were administered every 3 to 4 weeks in accordance with the approved dosing schedules and institutional clinical practice. The anti-PD-1 agents used in this study included sintilimab (200 mg, n = 7), tislelizumab (200 mg, n = 6), toripalimab (240 mg, n = 4), camrelizumab (200 mg, n = 3), and nivolumab (240 mg, n = 3), all of which were delivered intravenously. For patients receiving combination therapy, ICIs were administered for 4–6 cycles, which was identical to and synchronized with the concurrent chemotherapy backbone within the same treatment cycle. Treatment with ICIs or chemotherapy was continued until disease progression, unacceptable toxicity, or treatment discontinuation based on the physician’s judgment. Specifically, grade 3–4 adverse events (AEs), such as hypertension, aspartate aminotransferase elevation, upper gastrointestinal bleeding, or other serious toxic effects, were considered indications for treatment modification or discontinuation.

### 2.3 Baseline characteristics and clinical outcomes

Baseline clinicopathological data were systematically collected and analyzed for all patients prior to the initiation of therapy. Tumor therapeutic efficacy was evaluated in accordance with the Response Evaluation Criteria in Solid Tumors (RECIST) version 1.1. All radiological evaluations were investigator-assessed by two independent, experienced radiologists/oncologists who were blinded to the patients’ clinical group assignments. Any discrepancies in tumor response assessment were resolved through consensus or arbitration by a third senior specialist. The primary endpoint of this study was overall survival (OS), which was defined as the interval from the date of initial treatment following recurrence to the date of death from any cause, or to the date of the last follow-up for patients who were alive. Secondary endpoint was progression-free survival (PFS), which ascertained as the time from the first treatment for recurrence to clinical or radiological disease progression or death. Objective response rate (ORR) was calculated as the proportion of patients achieving either a complete response (CR) or a partial response (PR). Disease control rate (DCR) was defined as the percentage of patients who demonstrated CR, PR, or stable disease (SD). Treatment-related toxicities and AEs were monitored and graded according to the National Cancer Institute Common Terminology Criteria for Adverse Events (NCICTCAE) version 5.0. The data cutoff for the final follow-up was July 31, 2024.

### 2.4 Statistical analysis

Statistical evaluations were performed using R software (version 4.4.3). Categorical variables were presented as frequencies and percentages, and comparisons between groups were conducted using Pearson’s chi-square test or Fisher’s exact test, as appropriate. For continuous variables, data were presented as mean ± standard deviation (for parametric distributions) or median with interquartile range (IQR) (for non-parametric distributions), and compared using Student’s t-test or the Mann-Whitney U test. The proportional hazards assumption for the Cox regression models was rigorously verified prior to survival analysis using both graphical methods and formal statistical tests based on Schoenfeld residuals. To reduce selection bias and potential confounding inherent in this retrospective study, inverse probability of treatment weighting (IPTW) was performed. Propensity scores were calculated using a logistic regression model based on three clinically relevant baseline covariates: CA19–9 level, peritoneal recurrence, and postoperative adjuvant chemotherapy. Stabilized weights were then calculated for each patient. The balance of baseline characteristics between the two groups before and after weighting was evaluated using the standardized mean difference (SMD).

In both the unweighted and IPTW-weighted cohorts, OS and PFS were estimated employing the Kaplan-Meier method and analyzed via the log-rank test. OS and PFS were estimated employing the Kaplan-Meier method and analyzed via the log-rank test. Furthermore, univariate and multivariate Cox proportional hazards regression models were utilized to determine hazard ratios (HRs) and their corresponding 95% confidence interval (CI) to identify significant prognostic factors. A two-sided *P* < 0.05 was considered statistically significant.

## 3 Results

### 3.1 Patient characteristics

A total of 113 patients with recurrent PC met the inclusion criteria, including 23 in the chemotherapy combined with ICIs group and 90 in the chemotherapy group. Demographics and clinical characteristics are shown in **Table 1**. Among the 113 patients, the median age was 59 years (range: 54-67), and 60 patients (53.1%) were male. Most patients had an ECOG PS of 0 (n = 101, 89.4%). The primary tumor was predominantly located in the pancreatic head (n = 73, 64.6%), and 72 patients (63.7%) had tumor measuring ≤ 40mm, with a mean tumor diameter of 40.0mm (29.0-50.0mm). Poorly differentiated tumors were observed in 67 patients (59.3%). Pathological lymph node metastasis, microvascular invasion, and perineural invasion were identified in 53 (46.9%), 47 (41.6%), and 101 (89.4%) patients, respectively. According to the AJCC 8^th^ edition staging system, 88 patients (77.9%) had stage I/II disease at initial diagnosis. In terms of recurrence pattern, liver metastasis was the most common site of progression (n = 48, 42.5%), followed by distant lymph node metastasis (n = 34, 30.1%) and peritoneal recurrence (n = 27, 23.9%). The baseline characteristics of the two groups were generally balanced (P > 0.05). To further adjust for potentially unrecognized selection bias, an IPTW analysis was performed. As detailed in **Supplementary Table 1**, the groups achieved an excellent balance across the pre-specified, clinically relevant confounders.

**Table 1:** Patients and disease characteristics according to treatment group.

Variable	Chem + ICIs	Chem	*P*
	(n = 23)	(n = 90)	
Age, years			0.264
≤ 60	15 (65.2)	47 (52.2)	
> 60	8 (34.8)	43 (47.8)	
Gender			0.712
Male	13 (56.5)	47 (52.2)	
Female	10 (43.5)	43 (47.8)	
ECOG PS			0.475
0	22 (95.7)	79 (87.8)	
≥ 1	1 (4.3)	11 (12.2)	
Tumor location			0.282
Head	14 (60.9)	59 (65.6)	
Body/Tail	9 (39.1)	31 (34.4)	
Tumor size (mm)			0.867
≤ 40	15 (65.2)	57 (63.3)	
> 40	8 (34.8)	33 (36.7)	
Differentiation			0.675
Well/moderate	10 (43.5)	36 (40.0)	
Poor	13 (56.5)	54 (60.0)	
Lymph node metastases			0.192
No	15 (65.2)	45 (50.0)	
Yes	8 (34.8)	45 (50.0)	
TNM stage			0.282
I/II	16 (69.6)	72 (80.0)	
III/IV	7 (30.4)	18 (20.0)	
Progressive-pattern			
Local	1 (4.3)	6 (6.7)	1.000
Liver	7 (30.4)	41 (45.6)	0.190
Lung	1 (4.3)	5 (5.6)	1.000
Distant lymph node	4 (17.4)	30 (33.3)	0.137
Peritoneal	9 (39.1)	18 (20.0)	0.055
Other	4 (17.4)	7 (7.8)	0.320
Microvascular invasion			0.837
No	13 (56.5)	53 (58.9)	
Yes	10 (43.5)	37 (41.1)	
Perineural invasion			1.000
No	2 (8.7)	10 (11.1)	
Yes	21 (91.3)	80 (88.9)	
CA19-9 (U/ml)			0.095
< 35	12 (52.2)	30 (33.3)	
≥ 35	11 (47.8)	60 (66.7)	
CEA (ng/ml)			0.219
< 5	13 (56.5)	38 (42.2)	
≥ 5	10 (43.5)	52 (57.8)	
CA12-5 (U/ml)			0.968
< 35	16 (72.7)	63 (70.0)	
≥ 35	7 (30.4)	27 (30.0)	
Adjuvant chemotherapy			0.208
No	5 (21.7)	32 (35.6)	
Yes	18 (78.3)	58 (64.4)	
WBC (×10^9^/l)			0.819
≤ 100	20 (87.0)	74 (82.2)	
> 100	3 (13.0)	16 (17.8)	
HGB (g/l)			0.513
< 130	16 (69.6)	56 (62.2)	
≥ 130	7 (30.4)	34 (37.8)	
PLT (×10^9^/l)			1.000
≤ 100	1 (4.3)	2 (2.2)	
> 100	22 (95.7)	88 (97.8)	
ALT (U/L)			0.727
≤ 40	20 (87.0)	73 (81.1)	
> 40	3 (13.0)	17 (18.9)	
AST (U/L)			0.433
≤ 40	18 (78.3)	63 (70.0)	
> 40	5 (21.7)	27 (30.0)	
ALB (g/L)			0.172
≤ 40	3 (13.0)	24 (26.7)	
> 40	20 (87.0)	66 (73.3)	
TBIL (μmol/l)			1.000
≤ 20.5	19 (82.6)	76 (84.4)	
> 20.5	4 (17.4)	14 (15.6)	
ALBI grade			0.116
1	22 (95.7)	71 (78.9)	
2 or 3	1 (4.3)	19 (21.1)	

ECOG PS, Eastern Cooperative Oncology Group performance status; TNM, tumor-node-metastases stage; CA 19-9, carbohydrate antigen 19-9; CEA, carcinoembryonic antigen; CA 125, carbohydrate antigen 125; WBC, white blood cell count; HGB, hemoglobin; PLT, platelet count; ALT, alanine transaminase; AST, aspartate aminotransferase; ALB, albumin; TBIL, total bilirubin; ALBI, albumin-bilirubin.

### 3.2 Survival outcomes

**Figure 2:**
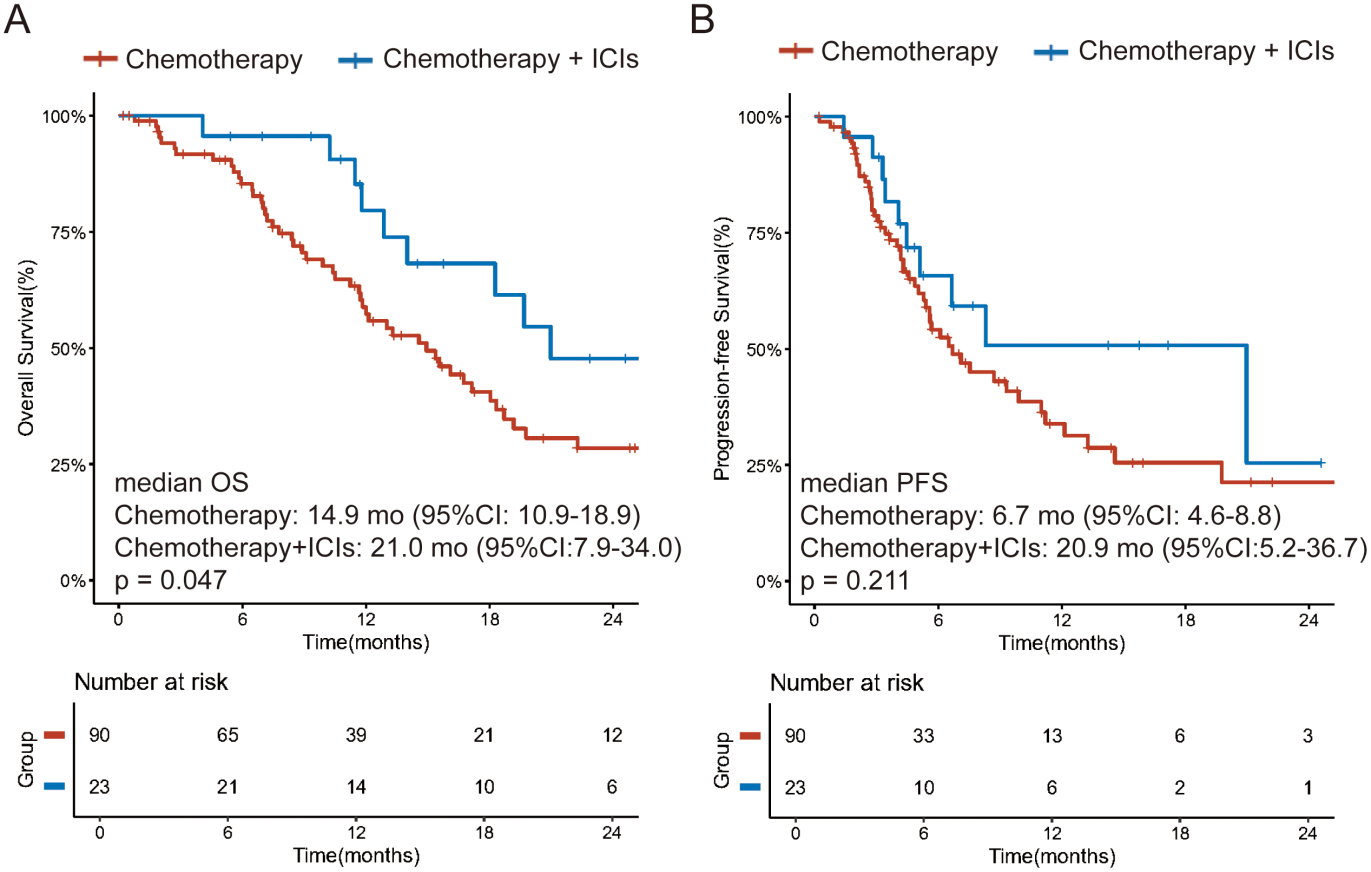
Kaplan-Meier survival curves comparing OS **(A)** and PFS **(B)** among eligible patients. who underwent chemotherapy combined with ICIs group versuschemotherapy group. OS, overall survival; PFS, progression-free survival; ICIs, Immune checkpoint inhibitors.

The median follow-up duration for the entire cohort was 22.8 months (range 16.8-29.0 months). For all patients, the median OS was 16.1 months (95% CI: 12.5-19.7), with estimated 1-, 2-, and 3-year survival rates of 62.2%, 32.5%, and 27.8%, respectively. Survival analysis showed that patients in the chemotherapy combined with ICIs group had significantly better OS than those in the chemotherapy alone group. The median OS was 21.0 months (95% CI: 7.9-34.0) in the chemotherapy combined with ICIs group, compared with 14.9 months (95% CI: 10.9-18.9) in the chemotherapy group (HR = 0.508 [95% CI: 0.257-1.002]; log-rank *P* = 0.047; Cox *P* = 0.051; **Figure 2A**). Consistently, the 1-, 2-, and 3-year OS rates were higher in the chemotherapy combined with ICIs group than in the chemotherapy group (79.6%, 47.8%, and 38.2% vs. 57.3%, 28.4%, and 25.6%, respectively). For PFS, the median PFS was 20.9 months (95% CI: 5.2-36.7) in the chemotherapy combined with ICIs group and 6.7 months (95% CI: 4.6-8.8) in the chemotherapy group (HR = 0.917 [95% CI: 0.463-1.814]; Cox *P* = 803; **Figure 2B**). Although a numerically longer PFS was observed in the chemotherapy combined with ICIs group, the difference between the two groups did not reach statistical significance (*P* = 0.211). The 1-year PFS rate was also higher in the chemotherapy combined with ICIs group than in the chemotherapy group (50.7% vs. 31.3%).

**Figure 3:**
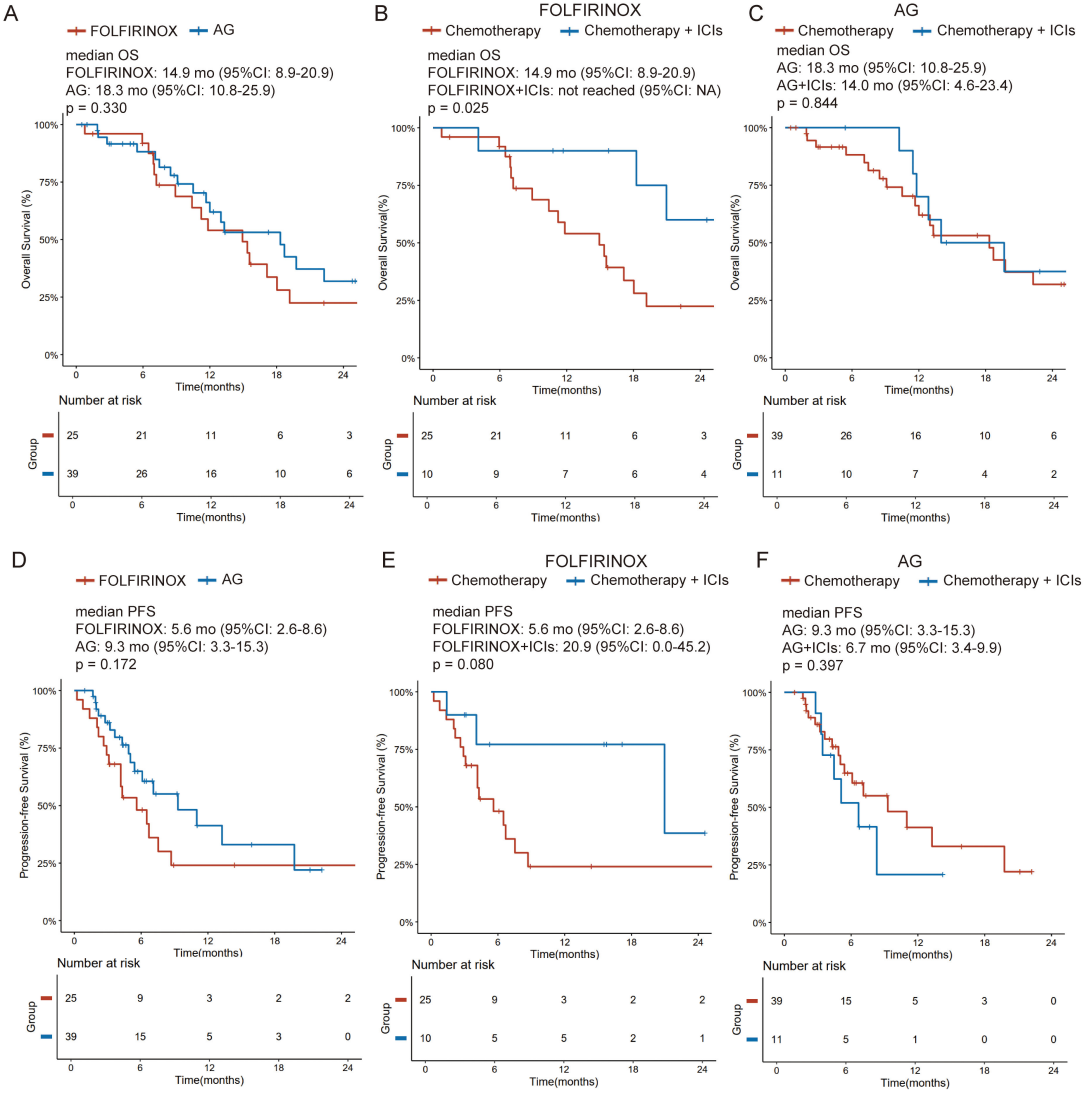
Kaplan-Meier survival curves comparing OS and PFS according to treatment regimens. **(A ,D)** Survival outcomes of patients treated with the FOLFIRINOX regimen versus the AG regimen. **(B, E)** Comparison between FOLFIRINOX combined with ICIs and FOLFIRINOX alone. **(C, F)** Comparison between AG combined with ICIs and AG alone. OS, overall survival; PFS, progression-free survival; ICIs, Immune checkpoint inhibitors; AG, Gemcitabine plus Nab-paclitaxel; FOLFIRINOX, 5-fluorouracil, leucovorin, irinotecan, and oxaliplatin.

The distribution of different treatment strategies within the study cohort was illustrated in **Supplementary Figure 1A**. For different chemotherapy, no significant difference in OS was observed between patients receiving AG and those receiving FOLFIRINOX as chemotherapy alone, with median OS of 18.3 months (95% CI: 10.8-25.9) and 14.9 months (95% CI: 8.9-20.9), respectively (*P* = 0.330, **Figure 3A**). Among patients treated with FOLFIRINOX, the FOLFIRINOX plus ICIs group was associated with longer OS compared with the chemotherapy alone group (median OS: not reached [95% CI: NA] vs. 14.9 months [95% CI: 8.9-20.9]; HR = 0.266 [95% CI: 0.077-0.919]; log-rank *P* = 0.025; Cox *P =* 0.036; **Figure 3B**). However, among patients receiving AG, the addition of ICIs did not significantly improve OS compared with AG alone, with a median OS of 14.0 months (95% CI: 4.6-23.4) in the combination group versus 18.3 months (95% CI: 10.8-25.9) in the monotherapy group (HR = 0.915 [95% CI: 0.378-2.219]; log-rank *P* = 0.844; Cox *P* = 0.845; **Figure 3C**).

With respect to PFS, no significant difference was identified between the two chemotherapy regimens when administered alone, with median PFS of 9.3 months (95% CI: 3.3-15.3) for AG and 5.6 months (95% CI: 2.6-8.6) for FOLFIRINOX (*P* = 0.172, **Figure 3D**). For patients receiving FOLFIRINOX, the FOLFIRINOX plus ICIs group demonstrated a trend toward improved PFS compared with the chemotherapy alone group, with median PFS of 20.9 months (95% CI: 0.0-45.2) versus 5.6 months (95% CI: 2.6-8.6; HR = 0.347 [95% CI: 0.100-1.202]; log-rank *P* = 0.080; Cox *P* = 0.095; **Figure 3E**). In contrast, for patients receiving AG, the addition of ICIs did not confer a PFS benefit over AG alone, with median PFS of 6.7 months (95% CI: 3.4-9.9) versus 9.3 months (95% CI: 3.3-15.3; HR = 1.468 [95% CI: 0.600-3.593]; log-rank *P* = 0.397; Cox *P* = 0.400; **Figure 3F**). The addition of ICIs to the chemotherapy backbone failed to demonstrate a significant clinical advantage. Apart from the FOLFIRINOX and AG cohorts, no statistically significant differences were observed between patients receiving other chemotherapy alone and those receiving other chemotherapy combined with ICIs (*P* > 0.05, **Supplementary Figures 1B, C**).

In the IPTW-weighted cohort, survival outcomes remained highly consistent with the primary unweighted analysis, confirming that our core conclusions were stable. Specifically, within the weighted overall population, patients in the chemotherapy combined with ICIs group achieved a statistically significant improvement in OS compared with those in the chemotherapy group (median OS:30.6 [95% CI: 18.3-NA] vs. 15.4 months [95% CI: 11.8-19.2]; log-rank *P* = 0.006; HR = 0.436 [95% CI: 0.224-0.850], Cox *P* = 0.015; **Supplementary Figure 2A**). However, no statistically significant difference was detected for PFS between the two groups in the weighted total population (*P* = 0.081, **Supplementary Figure 2E**). For the two chemotherapy backbones administered alone without ICIs, no significant survival differences were found between the FOLFIRINOX group and the AG group regarding either OS (*P* = 0.284, **Supplementary Figure 2B**) or PFS (*P* = 0.201, **Supplementary Figure 2F**). Crucially, subgroup analyses within the IPTW-weighted cohort confirmed that the survival benefit of adding ICIs was uniquely tied to the FOLFIRINOX backbone. Among patients receiving FOLFIRINOX, the addition of ICIs was significantly associated with longer OS (median OS: NA [95% CI: 21.0-NA] vs. 14.9 months (95% CI: 10.4-NA); *P* = 0.003, **Supplementary Figure 2C**) and longer PFS (median PFS: NA [95% CI: 21.0-NA] vs. 5.6 months (95% CI: 4.2-NA); *P* = 0.003, **Supplementary Figure 2G**) compared to FOLFIRINOX alone. Conversely, for patients on the AG regimen, combining ICIs with chemotherapy provided no additional benefit over AG alone for either OS (*P* = 0.590, **Supplementary Figure 2D**) or PFS (*P* = 0.469, **Supplementary Figure 2H**). Taken together, these doubly robust IPTW findings indicated that the superior efficacy observed with the FOLFIRINOX plus ICIs combination strategy was statistically sound and not driven by baseline clinical confounders.

### 3.3 Treatment efficacy

**Figure 4:**
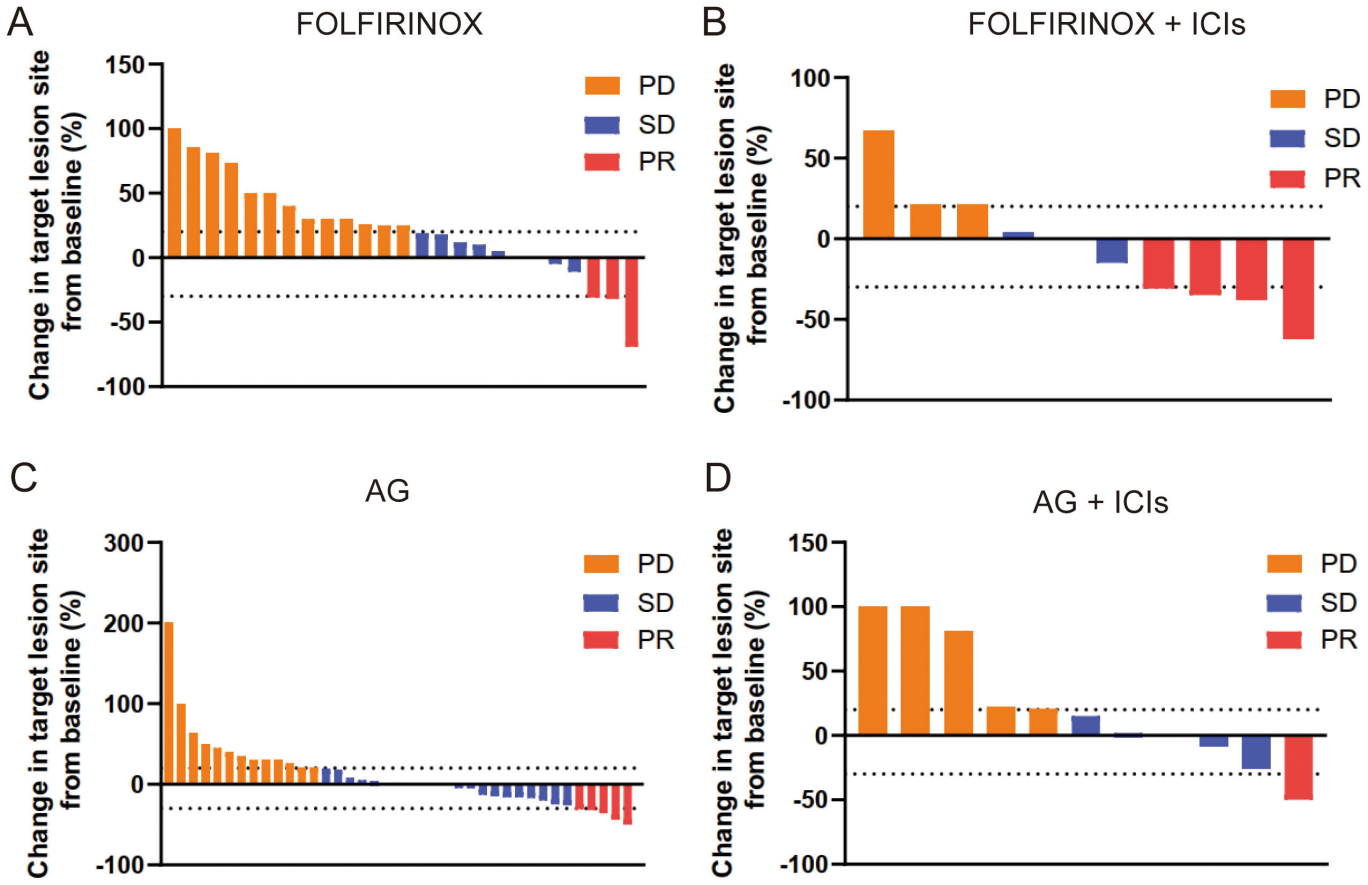
Distribution of best objective responses across different treatment cohorts. 100% stacked bar charts illustrating the proportion of patients achieving Partial Response (PR), Stable Disease (SD), and Progressive Disease (PD) according to RECIST version 1.1 criteria. **(A)** Clinical response in patients receiving FOLFIRINOX chemotherapy alone. **(B)** Clinical response in patients receiving FOLFIRINOX combined with ICIs. **(C)** Clinical response in patients receiving AG alone. **(D)** Clinical response in patients receiving AG combined with ICIs. ICIs, immune checkpoint inhibitors; AG, Gemcitabine plus Nab-paclitaxel; FOLFIRINOX, 5-fluorouracil, leucovorin, irinotecan, and oxaliplatin; RECIST, Response Evaluation Criteria in Solid Tumors.

The waterfall plots illustrated the best percentage change in target lesions according to RECIST criteria. Among patients receiving FOLFIRINOX alone, a total of 25 patients were evaluable, of whom 13 had progressive disease (PD), 9 had SD, and 3 achieved PR, resulting in an ORR of 12.0% and a DCR of 48% (**Figure 4A**). For those treated with FOLFIRINOX plus ICI, among 10 evaluable patients, 3 had PD, 3 had SD, and 4 achieved PR, with an ORR of 40.0% and a DCR of 70.0% (**Figure 4B**). In patients receiving AG alone, among 39 evaluable patients, 13 achieved a best response of PD, 5 achieved PR, and the remaining were classified as SD, yielding an ORR of 12.8% and a DCR of 66.7% (**Figure 4C**). For patients treated with AG in combination with ICI, among 11 evaluable patients, 5 had PD, 5 had SD, and 1 achieved PR, with corresponding ORR of 9.1% and DCR of 54.5% (**Figure 4D**). These radiographic response patterns were consistent with the observed survival trends, particularly in the FOLFIRINOX-based subgroup where the addition of ICI was associated with numerically higher response rates.

### 3.4 Prognostic factor analysis

As shown in **Table 2**, both univariate and multivariate analyses identified local recurrence and treatment strategy as significant prognostic factors for OS. In the univariate analysis, local recurrence was associated with a HR of 3.36 (95% CI: 1.33-8.49, *P* = 0.011), and the combination group was associated with an HR of 0.51 (95% CI: 0.26-1.00, *P* = 0.036) compared to the chemotherapy group. In the multivariate analysis, local recurrence remained a significant factor (HR: 4.73, 95% CI: 1.74-12.82, *P* = 0.002), as did treatment strategy (HR: 0.48, 95% CI: 0.24-0.96, *P* = 0.037), underscoring the independent prognostic value of treatment strategy for OS. Regarding PFS, univariable analysis revealed that local recurrence (HR: 2.88, 95% CI: 1.14-7.33; *P* = 0.026), liver recurrence (HR: 1.80, 95% CI:1.10-3.00, *P* = 0.022), and other recurrence (HR: 0.20, 95% CI:0.05-0.82; *P* = 0.026) were significantly associated with PFS. In the multivariate Cox proportional hazards model, local recurrence was identified as the only independent predictor of PFS (HR: 3.71, 95% CI: 1.22-11.27, *P* = 0.021).

**Table 2:** Univariate and multivariate analysis for OS and PFS.

Variable	OS				PFS			
	Univariate analysis		Multivariate analysis		Univariate analysis		Multivariate analysis	
	HR (95% CI)	*P*	HR (95% CI)	*P*	HR (95% CI)	*P*	HR (95% CI)	*P*
Treatment (Chem+ICIs/Chem)	0.51 (0.26-1.00)	0.036	0.48 (0.24-0.96)	0.037	0.65 (0.33-1.28)	0.211	0.77 (0.39-1.53)	0.454
Age, years (> 60, ≤ 60)	1.00 (0.62-1.70)	0.900			0.89 (0.54-1.50)	0.670		
Gender (Male, Female)	1.50 (0.91-2.50)	0.110			1.50 (0.89-2.50)	0.130		
ECOG PS (≥1, 0)	0.98 (0.45-2.20)	0.960			0.87 (0.39-1.90)	0.730		
Tumor size, mm (>40, ≤ 40)	0.67 (0.38-1.20)	0.170			0.61 (0.35-1.10)	0.088		
Differentiation (Poor, Well/moderate)	1.10 (0.66-1.90)	0.690			1.40 (0.86-2.40)	0.160		
Lymph node metastases (Yes, No)	1.60 (0.94-2.60)	0.083			1.50 (0.89-2.50)	0.130		
TNM stage (III/IV, I/II)	1.30 (0.68-2.40)	0.450			1.10 (0.61-2.1)	0.680		
Local recurrence (Yes, No)	3.36 (1.33-8.49)	0.011	4.73 (1.74-12.81)	0.002	2.88 (1.14-7.33)	0.026	3.71 (1.22-11.27)	0.021
Liver recurrence (Yes, No)	1.60 (0.96-2.60)	0.069	1.48 (0.85-2.58)	0.161	1.80(1.10-3.00)	0.022	1.77 (0.86-3.64)	0.118
Lung recurrence (Yes, No)	0.29 (0.07-1.20)	0.086	0.35 (082-1.51)	0.161	0.44 (0.11-1.80)	0.250	0.69 (0.16-2.98)	0.619
Lymph node recurrence (Yes, No)	0.80 (0.45-1.40)	0.450			0.90 (0.50-1.60)	0.720		
Peritoneal recurrence (Yes, No)	1.10 (0.62-2.00)	0.720			0.48 (0.22-1.10)	0.081	1.57 (0.72-3.41)	0.253
Other recurrence (Yes, No)	0.27 (0.07-1.10)	0.071			0.20 (0.05-0.82)	0.026	3.92 (1.31-11.70)	0.138
Microvascular invasion (Yes, No)	1.20 (0.70-2.00)	0.540			1.00 (0.60-1.70)	0.990		
Perineural invasion (Yes, No)	1.10 (0.62-2.00)	0.600			0.48 (0.22-1.10)	0.950		
CA19-9, U/ml (≥ 35, <35)	1.10 (0.63-1.80)	0.830			1.10 (0.63-1.80)	0.820		
CEA, ng/ml (≥ 5, <5)	1.40 (0.82-2.30)	0.220			1.60 (0.93-2.60)	0.094		
CA12-5, U/ml (≥ 35, <35)	1.70 (0.97-2.90)	0.083			1.20 (0.68-2.10)	0.540		
Adjuvant chemotherapy (Yes, No)	1.10 (0.65-1.90)	0.720			0.98 (0.57-1.70)	0.950		
TBIL, μmol/l (>20.5, ≤ 20.5)	1.10 (0.55-2.20)	0.790			1.00 (0.52-2.00)	0.940		

ECOG PS, Eastern Cooperative Oncology Group performance status; TNM, tumor-node-metastases stage; CA 19-9, carbohydrate antigen 19-9; CEA, carcinoembryonic antigen; CA 125, carbohydrate antigen 125; TBIL, total bilirubin.

### 3.5 Adverse events

**Figure 5:**
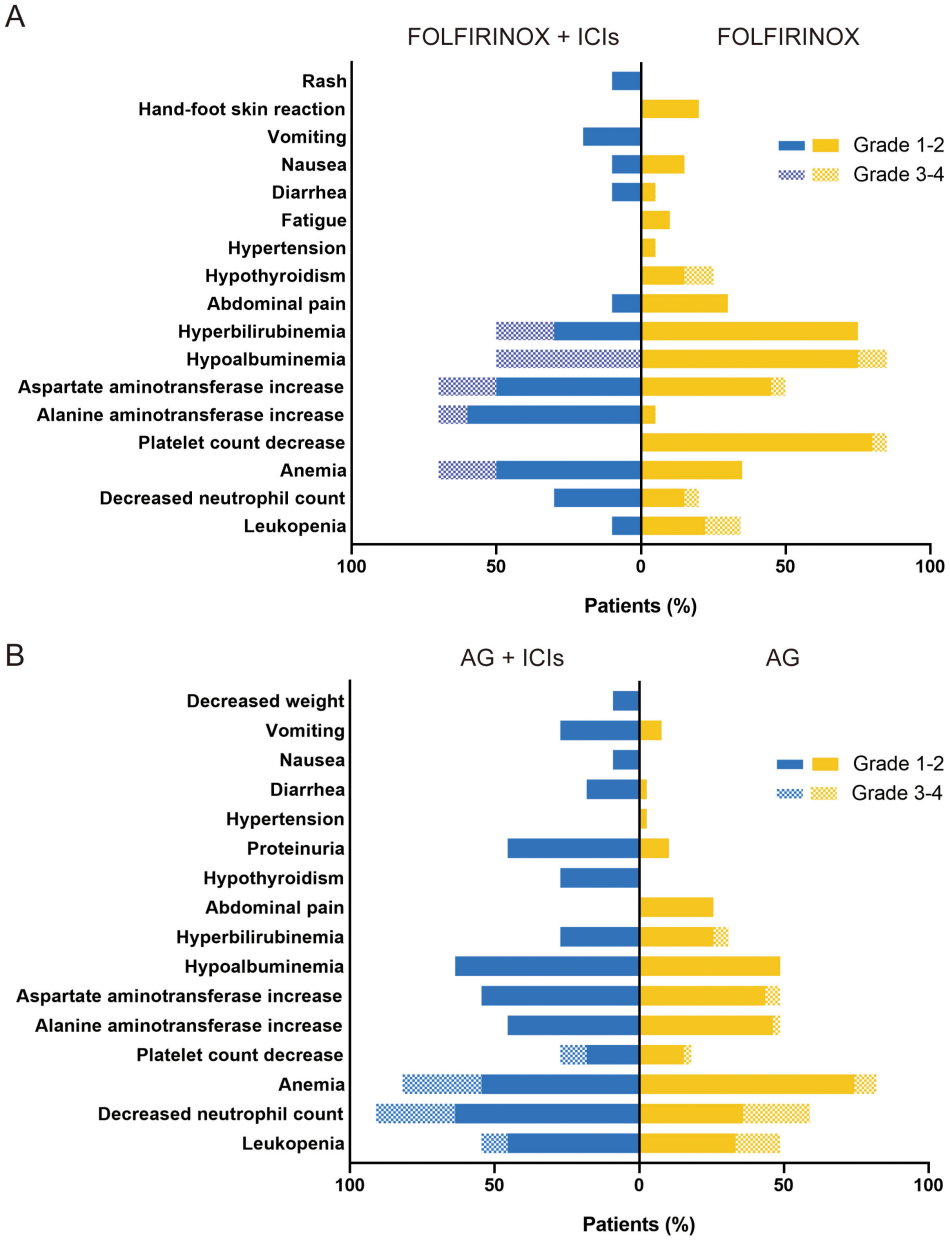
Comparison of treatment-related AEs across treatment cohorts. Side-by-side bar charts illustrating the incidence and severity of common AEs (all grades) categorized by chemotherapy backbone and the addition of ICIs. **(A)** Comparison of the safety profile between FOLFIRINOX combined with ICIs and FOLFIRINOX alone. **(B)** Comparison of the safety profile between AG combined with ICIs and AG alone. ICIs, immune checkpoint inhibitors; AG, Gemcitabine plus Nab-paclitaxel; FOLFIRINOX, 5-fluorouracil, leucovorin, irinotecan, and oxaliplatin; AEs, adverse events.

A comprehensive subgroup analysis was performed to evaluate the safety of chemotherapy combined with ICIs and chemotherapy. The comparative incidences of treatment-related AEs between the FOLFIRINOX plus ICIs cohort and the FOLFIRINOX cohort were depicted in **Figure 5A**. No statistically significant differences were observed in the overall incidence of adverse events between the two cohorts, with the notable exception of Grade 3–4 hyperbilirubinemia. Specifically, the frequency of severe hyperbilirubinemia was significantly higher in the FOLFIRINOX plus ICIs group compared to the FOLFIRINOX-only group (20.0% vs. 0.0%, *P* = 0.021), while other toxicities remained statistically comparable (*P* > 0.05). For AG regimen, the safety profiles were generally balanced across both arms, except for Grade 1–2 Proteinuria occurred in 45.5% of patients receiving AG plus ICIs, whereas no such events were reported in the AG monotherapy cohort (*P* = 0.017, **Figure 5B**). Incidence rates for all other treatment-related adverse events showed no significant intergroup disparity (**Supplementary Table 2**).

## 4 Discussion

Despite continuous advances in radical surgery and adjuvant chemotherapy, as many as 65%-75% of patients with PC still develop locoregional recurrence or distant metastasis within 5 years after surgery (27). At present, no standard therapeutic strategy has been established for recurrent PC, and salvage treatment in routine clinical practice still largely relies on systemic chemotherapy, with only limited survival benefit (4, 28, 29). Against this background, the present study was conducted to evaluate the efficacy and safety of chemotherapy combined with ICIs in patients with recurrent PC. Importantly, our analysis further dissected the chemotherapy backbone and specifically compared the performance of the two major regimens, AG and FOLFIRINOX, when combined with immunotherapy. Our results showed that FOLFIRINOX plus ICIs achieved superior antitumor activity compared with FOLFIRINOX alone, with an objective response rate of approximately 40%, without an apparent increase in treatment-related toxicity. In contrast, the addition of ICIs to the AG regimen did not confer a clear additional benefit. To our knowledge, this is the first retrospective study in the recurrent PC setting to suggest that the efficacy of chemoimmunotherapy may differ substantially according to the chemotherapy backbone.

These findings are clinically meaningful because they challenge the conventional view that systemic chemotherapy can be regarded as a biologically uniform partner for immunotherapy (30). In recent years, immunotherapy as monotherapy has shown limited efficacy in pancreatic cancer, largely because this disease is typically associated with an immunologically “cold” TME (12). This TME is characterized by dense desmoplastic stroma, extensive infiltration of myeloid cells, scarce CD8^+^ T-cell infiltration, and low expression of immune activation markers such as granzyme B and interferon-gamma. In addition, chronic inflammation within the PC microenvironment paradoxically promotes immunosuppression rather than effective antitumor immunity, thereby facilitating immune escape and tumor progression (19, 31). These features indicate either an absence of functional adaptive T-cell responses or an intrinsic resistance to immune-based therapies. However, recurrent lesions differ fundamentally from treatment-naive tumors in both biological and immunological characteristics (32, 33). Emerging studies on tumor evolution have shown that, under the strong selective pressure imposed by surgery and systemic therapy, recurrent tumors often undergo a marked genetic bottleneck, accompanied by increased mutational burden and enhanced subclonal heterogeneity (30). Such treatment-driven clonal evolution may result in the generation of more neoantigens (16), which might confer a potentially greater degree of immunogenicity than that observed in newly diagnosed disease. Nevertheless, this potentially latent immune potential may still require an appropriate external trigger to be fully activated (28). In this context, the markedly different outcomes observed between FOLFIRINOX- and AG-based chemoimmunotherapy in our study provide intriguing clinical support to hypothesize that not all chemotherapy regimens are equally capable of sensitizing recurrent pancreatic tumors to ICIs. While we did not directly evaluate TME alterations or neoantigen dynamics in this clinical cohort, our results tentatively underscore the importance of precisely selecting the chemotherapy backbone rather than combining immunotherapy indiscriminately with any cytotoxic regimen.

From a mechanistic perspective, the differential efficacy observed in our study may be related to the distinct immunomodulatory properties of the two regimens. FOLFIRINOX contains oxaliplatin, which has been reported to induce immunogenic cell death, promote antigen release, and enhance antitumor immune priming, thereby potentially creating a more favorable context for immune checkpoint blockade (34). By contrast, the AG regimen may differ in its capacity to remodel the tumor immune microenvironment and may therefore provide less effective synergy with ICIs in the recurrent setting. Although the precise mechanisms underlying these observations remain to be clarified, our findings suggest that the therapeutic benefit of chemoimmunotherapy in recurrent PC depends not only on the addition of ICIs, but also on the biological interaction between immunotherapy and the selected chemotherapy backbone (13, 20). In this regard, exploratory use of FOLFIRINOX plus ICIs may represent a particularly promising salvage strategy for selected patients with recurrent PC.

To contextualize our findings within the evolving immunotherapy landscape of PDAC, it is essential to consider prior milestone chemoimmunotherapy trials, many of which have yielded mixed or disappointing results. For instance, large-scale studies evaluating the addition of ICIs to standard gemcitabine-based backbones, such as the CCTG PA.7 trial (35), demonstrated that unselected PDAC patients rarely achieve a definitive survival benefit from immunotherapy. These historical negatives align tightly with our own observations in the AG subgroup. However, our study offers a notable contrast within the FOLFIRINOX subgroup. This systemic comparison within the current literature suggests that the failure of prior PC immunotherapy trials might not be a definitive indictment of ICIs themselves, but rather a reflection of a sub-optimal chemotherapy partner. This is strongly corroborated by the OPTIMIZE-1 trial, which demonstrated encouraging efficacy by combining the CD40 agonist mitazalimab with an mFOLFIRINOX backbone in advanced PC (36). Our findings thus provide a focus toward FOLFIRINOX as a choice for immune-checkpoint evaluation in recurrent PC.

Another noteworthy finding of this study concerns biomarker interpretation. Most patients in the chemotherapy combined with ICIs group who underwent genomic testing were found to have microsatellite-stable (MSS) disease and low tumor mutational burden (TMB-L), yet they still achieved meaningful survival benefit from FOLFIRINOX plus ICIs. This observation leads us to hypothesize that conventional biomarkers such as MSI status or overall tumor mutational burden (TMB) may be insufficient to fully capture the adaptive immunogenicity that emerges during tumor evolution, particularly when driven by local subclonal expansion in recurrent disease (30). We speculate that under the potent immunomodulatory effects of FOLFIRINOX, even tumors traditionally considered “cold” may be converted into a more immune-responsive state. This also implies that future clinical studies should move beyond static baseline biomarkers and incorporate more sensitive approaches, such as dynamic circulating tumor DNA (ctDNA) monitoring, to better characterize the evolving immune landscape of recurrent tumors and to identify patients most likely to benefit from chemoimmunotherapy. Further prospective trials with detailed multi-omic and longitudinal tissue analysis are warranted to validate these hypotheses.

In our cohort of 23 patients receiving ICIs combination therapy, 10 patients underwent genetic testing; among them, only one patient was microsatellite instability-high (MSI-H) and tumor mutational burden-high (TMB-H) status, while the remaining nine were MSS and TMB-L status. We acknowledge that comprehensive molecular profiling was unavailable for 43.5% (10/23) of ICI-treated patients, reflecting real-world resource constraints. However, this reflects the global patterns of PC patients accepting Next-Generation Sequencing at a low rate (37). The reason these MSS/TMB-L patients may benefit from ICIs was that chemotherapy may remodel the TME or induce Fc-mediated macrophage phagocytosis (38). Despite limited biomarker positivity, the entire ICIs cohort showed significant OS benefit than chemotherapy alone. This suggests that conventional biomarkers (MSI/TMB) may have limited sensitivity in cancer immunotherapy combined with chemotherapy selection (39, 40). This highlights the high immunological heterogeneity of PC. There is a need to identify more precise biomarkers to predict the effectiveness of immunotherapy. Although ICIs are not currently recommended in the NCCN guidelines, it can improve survival in some patients with pancreatic cancer, and this benefit may occur independently of MSI-H, dMMR, or TMB status (41, 42). In clinical practice in China, ICIs are recommended for some patients with recurrent pancreatic cancer based on the clinical judgment and experience of oncologists, with informed consent from the patients. Therefore, this study provides reference for the use of ICIs in the treatment of recurrent PC.

In the present study, compared with FOLFIRINOX alone, the addition of ICIs was associated with a numerically longer PFS, although the difference did not reach statistical significance, whereas OS was significantly improved in the FOLFIRINOX plus ICIs group. Several factors may explain the observed this discrepancy. First, the antitumor activity of ICIs is characterized by a temporal lag due to the complex kinetics of T-cell priming, clonal expansion, and microenvironmental remodeling. Unlike the rapid cytoreduction of chemotherapy, immune-mediated responses often manifest over months, potentially leading to a “tail effect” where long-term survival benefits exceed early radiographic improvements, a phenomenon well-documented in melanoma (43). Second, chemotherapy synergizes with ICIs by remodeling the TME. Chemotherapy-induced ICD triggers the release of DAMPs, promoting dendritic cell recruitment and antigen priming. Simultaneously, it depletes immunosuppressive Tregs and MDSCs, lowering the activation threshold for T cells. This dual mechanism creates a synergistic “priming effect” for ICIs. However, this biological restructuring requires a temporal window to manifest as radiographic changes. The inherent lag in immune-mediated remodeling often results in a delayed survival benefit, explaining why OS improvements frequently precede or exceed PFS gains in the recurrent setting. Third, conventional RECIST criteria may underestimate the clinical benefit of ICIs due to atypical response patterns like pseudoprogression and the durable “tail effect”, suggesting that dynamic biomarkers such as ctDNA may more accurately reflect therapeutic activity in recurrent PC than traditional radiography (44). Beyond these intrinsic biological mechanisms, several alternative explanations inherent to the retrospective nature of this study must also be considered when interpreting this clinical divergence. Post-progression treatments could confound the OS findings, as patients undergoing different subsequent salvage therapies might experience distinct survival prolongations after first-line progression. Additionally, differences in follow-up schedules and potential imbalances in censoring patterns between the two groups may influence the precise documentation of disease progression.

This study has several limitations. First, its retrospective design introduces inherent selection and indication biases. Second, the use of various ICIs led to treatment heterogeneity, potentially confounding the outcomes and limiting the generalizability of our findings. Third, the relatively small sample size, particularly in the chemo-immunotherapy cohort, constrained the statistical power and robustness of subgroup analyses. Despite these constraints, these real-world data provide valuable preliminary evidence for the synergistic potential of ICIs and chemotherapy in recurrent PC. Future prospective studies with larger, more uniform cohorts are warranted to validate these results and further elucidate the underlying biological mechanisms.

## 5 Conclusion

In conclusion, the efficacy of chemoimmunotherapy in recurrent PC may vary according to the chemotherapy backbone. Compared with FOLFIRINOX alone, FOLFIRINOX plus ICIs was associated with improved OS, although no significant difference in PFS was observed. In contrast, the addition of ICIs to the AG regimen did not confer a significant benefit in either OS or PFS. These findings indicate that FOLFIRINOX may represent a more suitable chemotherapy backbone for combination with immunotherapy in recurrent pancreatic cancer.

## Data Availability

The raw data supporting the conclusions of this article will be made available by the authors, without undue reservation.

## Supplementary material

Supplementary Figure 1 Distribution of therapeutic regimens and survival outcomes for other chemotherapy backbones. **(A)** Distribution of patients across different treatment strategies, including chemotherapy alone and their respective combinations with ICIs. **(B, C)** Kaplan-Meier curves comparing OS and PFS between patients receiving other chemotherapy regimens and those receiving other chemotherapy combined with ICIs. ICIs, immune checkpoint inhibitors; OS, overall survival; PFS, progression-free survival.

Supplementary Figure 2 Kaplan-Meier survival curves comparing OS and PFS according to treatment regimens after inverse probability of treatment weighting (IPTW). **(A, E)** Comparison of OS/PFS between the chemotherapy plus ICIs group and the chemotherapy alone group in the total population. **(B, F)** Survival outcomes of patients treated with the FOLFIRINOX regimen versus the AG regimen. **(C, G)** Comparison between FOLFIRINOX combined with ICIs and FOLFIRINOX alone. **(D, H)** Comparison between AG combined with ICIs and AG alone. OS, overall survival; PFS, progression-free survival; IPTW, inverse probability of treatment weighting; ICIs, Immune checkpoint inhibitors; AG, Gemcitabine plus Nab-paclitaxel; FOLFIRINOX, 5-fluorouracil, leucovorin, irinotecan, and oxaliplatin.
