# Supplementary material for: Efficacy of immune checkpoint inhibitors combined with different chemotherapy in recurrent pancreatic cancer: a retrospective study
Source: Front Immunol. 2026 Jul 30;17:1841783. doi: 10.3389/fimmu.2026.1841783 (PMC13468804; doi:10.3389/fimmu.2026.1841783)
Supplement: Supplementary file 1 [file Table1.docx]

**Supplementary Table 1. Patients and disease characteristics according to treatment group after IPTW.**

|  | Chem + ICIs | Chem | *P* | *SMD* |
| --- | --- | --- | --- | --- |
|  | (n = 111) | (n = 113) |  |  |
| Age, years |  |  | 0.281 | 0.285 |
| ≤ 60 | 74 (66.7) | 60 (53.1) |  |  |
| > 60 | 37 (33.3) | 53 (46.9) |  |  |
| Gender |  |  | 0.305 | 0.260 |
| Male | 72 (64.9) | 59 (52.2) |  |  |
| Female | 39 (35.1) | 54 (47.8) |  |  |
| ECOG PS |  |  | 0.256 | 0.296 |
| 0 | 106 (94.5) | 99 (87.6) |  |  |
| ≥ 1 | 5 (4.5) | 14 (12.4) |  |  |
| Tumor location | |  | 0.883 | 0.036 |
| Head | 75 (67.6) | 75 (66.4) |  |  |
| Body/Tail | 36 (32.4) | 38 (33.6) |  |  |
| Tumor size (mm) |  |  | 0.878 | 0.040 |
| ≤ 40 | 71 (63.9) | 70 (61.9) |  |  |
| > 40 | 40 (36.1) | 43 (38.1) |  |  |
| Differentiation |  |  | 0.649 | 0.115 |
| Well/moderate | 49 (44.1) | 44 (38.9) |  |  |
| Poor | 62 (55.9) | 69 (61.1) |  |  |
| Lymph node metastases | |  | 0.800 | 0.066 |
| No | 60 (54.1) | 58 (51.3) |  |  |
| Yes | 51 (45.9) | 55 (48.7) |  |  |
| TNM stage |  |  | 0.654 | 0.114 |
| I/II | 82 (73.9) | 89 (78.8) |  |  |
| III/IV | 29 (26.1) | 24 (21.2) |  |  |
| Progressive-pattern |  |  |  |  |
| Local | 5 (4.5) | 7 (6.2) | 0.690 | 0.098 |
| Liver | 37 (33.3) | 49 (43.4) | 0.418 | 0.215 |
| Lung | 9 (8.1) | 6 (5.3) | 0.704 | 0.104 |
| Distant lymph node | 20 (18.0) | 37 (32.7) | 0.219 | 0.343 |
| Peritoneal | 29 (26.1) | 27 (23.9) | 0.829 | 0.049 |
| Other | 20 (18.0) | 9 (8.0) | 0.181 | 0.308 |
| Microvascular invasion | |  | 0.373 | 0.226 |
| No | 54 (48.7) | 68 (60.2) |  |  |
| Yes | 57 (51.3) | 45 (39.8) |  |  |
| Perineural invasion |  |  | 0.491 | 0.160 |
| No | 7 (6.3) | 12 (10.6) |  |  |
| Yes | 104 (93.7) | 101 (89.3) |  |  |
| CA19-9 (U/ml) |  |  | 0.719 | 0.089 |
| < 35 | 46 (41.4) | 42 (37.1) |  |  |
| ≥ 35 | 65 (58.6) | 71 (62.8) |  |  |
| CEA (ng/ml) |  |  | 0.615 | 0.128 |
| < 5 | 56 (50.5) | 50 (44.2) |  |  |
| ≥ 5 | 55 (49.5) | 63 (55.8) |  |  |
| CA12-5 (U/ml) |  |  | 0.877 | 0.040 |
| < 35 | 78 (70.2) | 78 (69.0) |  |  |
| ≥ 35 | 33 (29.8) | 35 (31.0) |  |  |
| Adjuvant chemotherapy | |  | 0.979 | 0.005 |
| No | 7 (6.2) | 8 (7.1) |  |  |
| Yes | 103 (92.8) | 105 (92.9) |  |  |
| WBC (×10^9^/l) |  |  | 0.221 | 0.285 |
| ≤ 100 | 101 (91.0) | 93 (82.3) |  |  |
| > 100 | 10 (9.0) | 20 (17.7) |  |  |
| HGB (g/l) |  |  | 0.600 | 0.138 |
| < 130 | 75 (67.6) | 69 (61.1) |  |  |
| ≥ 130 | 36 (32.4) | 44 (38.9) |  |  |
| PLT (×10^9^/l) |  |  | 0.256 | 0.260 |
| ≤ 100 | 9 (8.1) | 2 (1.8) |  |  |
| > 100 | 102 (91.9) | 111 (98.2) |  |  |

**Supplementary Table 1.** Continued

|  | Chem + ICIs | Chem | *P* | *SMD* |
| --- | --- | --- | --- | --- |
|  | (n = 111) | (n = 113) |  |  |
| ALT (U/L) |  |  | 0.893 | 0.037 |
| ≤ 40 | 89 (80.2) | 93 (82.3) |  |  |
| > 40 | 22 (19.8) | 10 (8.9) |  |  |
| AST (U/L) |  |  | 0.899 | 0.034 |
| ≤ 40 | 80 (72.1) | 80 (70.8) |  |  |
| > 40 | 31 (27.9) | 33 (29.2) |  |  |
| ALB (g/L) |  |  | 0.118 | 0.397 |
| ≤ 40 | 11 (9.9) | 28 (24.8) |  |  |
| > 40 | 100 (90.1) | 85 (75.2) |  |  |
| TBIL (μmol/l) |  |  | 0.628 | 0.121 |
| ≤ 20.5 | 89 (80.2) | 96 (85.0) |  |  |
| > 20.5 | 22 (19.8) | 17 (15.0) |  |  |
| ALBI grade |  |  | 0.081 | 0.491 |
| 1 | 106 (95.5) | 91 (80.5) |  |  |
| 2 or 3 | 5 (4.5) | 22 (19.5) |  |  |

NOTE. IPTW, Inverse Probability of Treatment Weighting; ECOG PS, Eastern Cooperative Oncology Group performance status; TNM, tumor-node-metastases stage; CA 19-9, carbohydrate antigen 19-9; CEA, carcinoembryonic antigen; CA 125, carbohydrate antigen 125; WBC, white blood cell count; HGB, hemoglobin; PLT, platelet count; ALT, alanine transaminase; AST, aspartate aminotransferase; ALB, albumin; TBIL, total bilirubin; ALBI, albumin-bilirubin.

**Supplementary Table 2. Treatment-related adverse events before matching.**

| Adverse events, n (%) | **Any grade** | | |  | **Grade 3-4** | | |
| --- | --- | --- | --- | --- | --- | --- | --- |
|  | Chem +ICIs (n=23) | Chem (n=90) | *P* |  | Chem +ICIs (n=23) | Chem (n=90) | *P* |
| Leukopenia | 8 (34.8) | 31 (34.4) | 0.976 |  | 2 (8.7) | 11 (12.2) | 0.915 |
| Decreased neutrophil count | 14 (60.9) | 40 (44.4) | 0.241 |  | 4 (17.4) | 14 (15.6) | 1.000 |
| Anemia | 17 (73.9) | 69 (76.7) | 0.782 |  | 5 (21.7) | 6 (6.7) | 0.075 |
| Platelet count decrease | 4 (17.4) | 12 (13.3) | 0.870 |  | 2 (8.7) | 2 (2.2) | 0.386 |
| Alanine aminotransferase increase | 13 (56.5) | 39 (43.3) | 0.257 |  | 2 (8.7) | 2 (2.2) | 0.386 |
| Aspartate aminotransferase increase | 14 (60.9) | 48 (53.3) | 0.679 |  | 3 (13.0) | 5 (5.6) | 0.427 |
| Hypoalbuminemia | 13 (56.5) | 53 (58.9) | 0.837 |  | 0 | 0 | - |
| Hyperbilirubinemia | 9 (39.1) | 24 (26.7) | 0.241 |  | 3 (13.0) | 4 (4.4) | 0.297 |
| Abdominal pain | 4 (17.4) | 17 (18.9) | 1.000 |  | 0 | 2 (2.2) | 1.000 |
| Diarrhea | 3 (13.0) | 7 (7.8) | 0.702 |  | 0 | 0 | - |
| Hypothyroidism | 1 (4.3) | 1 (1.1) | 0.869 |  | 0 | 0 | - |
| Proteinuria | 5 (21.7) | 6 (6.7) | 0.075 |  | 0 | 0 | - |
| Hypertension | 0 | 2 (2.2) | 1.000 |  | 0 | 0 | - |
| Fatigue | 0 | 1 (1.1) | 1.000 |  | 0 | 0 | - |
| Decreased weight | 1 (4.3) | 0 | 0.460 |  | 0 | 0 | - |
| Nausea | 2 (8.7) | 1 (1.1) | 0.196 |  | 0 | 0 | - |
| Vomiting | 5 (21.7) | 6 (6.7) | 0.075 |  | 0 | 0 | - |
| Hand-foot skin reaction | 1 (4.3) | 1 (1.1) | 0.869 |  | 0 | 0 | - |
| Rash | 1 (4.3) | 0 | 0.460 |  | 0 | 0 | - |
